# Prior exposure to microplastics heightened the susceptibility of small intestine to radiation-induced injury in C57BL/6 mice

**DOI:** 10.1093/jrr/rraf046

**Published:** 2025-07-26

**Authors:** Jing Xu, Zhixing He

**Affiliations:** Department of Radiation Oncology, The Second Affiliated Hospital and Cancer Institute, Zhejiang University School of Medicine, No. 88 Jiefang Road, Shangcheng District, Hangzhou, 310009, China; Research Institute of Chinese Medical Clinical Foundation and Immunology, School of Basic Medical Science, Zhejiang Chinese Medical University, No. 548 Binwen Road, Binjiang District, Hangzhou‚ 310053, China

**Keywords:** microplastics, irradiation, intestinal injury, intestinal proteomics, pi3k signaling pathway

## Abstract

Microplastics (MPs) have been detected in multiple human organs, raising concerns about their potential health risks. The intestinal tract is particularly vulnerable to MPs exposure and accumulation. Radiotherapy often causes side effects such as radiation-induced intestinal injury (RIII). Although previous studies have shown that MPs exacerbate RIII by altering gut microbiota, their effect on the small intestine’s intrinsic sensitivity to radiation remains unclear. In this study, C57BL/6 mice were preexposed to MPs for a short period and then irradiated with 4 or 10 Gy to evaluate intestinal injury. Proteomic analysis of small intestine was performed to identify changes in protein expression. Short-term MPs exposure alone caused minimal intestinal damage. While 4 Gy irradiation did not cause significant intestinal injury, 10 Gy irradiation induced pronounced inflammation, increased epithelial apoptosis, and disrupted villus and lamina propria architecture. Importantly, the mice preexposed to MPs exhibited significantly increased sensitivity to RIII. Furthermore, prior MPs exposure significantly exacerbated RIII at 4 Gy but had no obvious influence on RIII at 10 Gy in C57BL/6 mice. The reason might be that the severe radiation-induced injury caused by 10 Gy could obscure the additional effects of prior MPs exposure. Proteomic analysis implicated the ‘PI3K-Akt signaling’ pathway as a key mediator of this effect. Indeed, treatment with a PI3K inhibitor could attenuate the MPs-driven susceptibility of small intestine to radiation. These findings underscore the need to minimize MPs exposure in patients undergoing radiotherapy.

## INTRODUCTION

Microplastics (MPs) pollution refers to plastic particles less than 5 mm in size, originating from the degradation of larger plastic debris, abrasion of plastic products, industrial activities, and the release of synthetic fibers during laundering [1]. Additionally, microbeads in personal care products contribute significantly to microplastic contamination. MPs can enter the human body through multiple pathways, including ingestion of contaminated food and water, inhalation of airborne particles, and exposure via certain consumer goods [2]. Once inside the body, microplastics have the potential to circulate through the bloodstream and accumulate in various tissues and organs. Research has identified the presence of MPs in human lungs [3], liver [4], kidneys [5], and even the placenta [6], prompting concerns about their potential impact on human health. While the field is still in its early stages, several possible mechanisms have been proposed linking microplastic exposure to various diseases, such as autoimmune disorders [7], Alzheimer’s disease [8], cancer [9], and asthma [10]. Despite substantial research on MPs and their associated health risks, additional studies are required to further elucidate the relationship between microplastics and other diseases.

The gut, as the primary interface for digestion and absorption, is particularly vulnerable to microplastic exposure [11]. Emerging research suggests a potential link between MPs exposure and the development of various gut diseases, including inflammatory bowel diseases [12] and colorectal cancer [13]. Several mechanisms, such as gut inflammation, barrier disruption, and gut microbiota imbalance, suggest that MPs may play a role in the development or exacerbation of gut-related diseases [14]. The above mechanisms are also known to cause intestinal damage by irradiation exposure [15, 16]. The potential for synergistic effects between radiation and MPs on intestinal injury is an area of growing concern.

Radiation, particularly ionizing radiation, is well known for its ability to cause damage to rapidly dividing cells, such as those in the intestinal epithelium [17]. Radiation-induced intestinal injury (RIII) is a common and serious side effect of radiotherapy for cancers in the abdominal and pelvic regions [18]. From acute gastrointestinal distress to chronic, life-altering conditions, the dangers of intestinal damage are far-reaching and can compromise both treatment outcomes and long-term health [19]. To better prevent, intervene, or manage this condition, it is crucial to understand the factors that influence the risk of developing radiation-induced intestinal damage, as well as those that may aggravate it. Understanding the factors that can aggravate or mitigate injury is essential for improving patient outcomes, ensuring that the benefits of cancer treatment outweigh the potential harm to healthy tissues.

The aim of this study is to investigate the effects of MPs exposure on RIII in C57BL/6 mice. It is hypothesized that MPs exposure may increase the vulnerability of intestinal tissues to radiation-induced damage in these mice. To explore this interaction, proteomic analysis of the small intestine was conducted to elucidate the complex effects of MPs exposure on RIII, as well as the underlying molecular mechanisms. The findings from this study enhance the understanding of MPs toxicity and highlight the critical importance of environmental conservation efforts.

## MATERIALS AND METHODS

### Animals and chemicals

Six-week-old male C57BL/6 mice were purchased from Shanghai SLAC Laboratory Animal Co., Ltd. Upon arrival, they were acclimated in the Zhejiang Chinese Medical University animal facility for 1 week, then randomly assigned to experimental groups. Mice were maintained under a 12-h light/dark cycle at 25 ± 1°C, and 50 ± 5% humidity, with ad libitum access to food and water. All procedures were approved by the Institutional Animal Care and Use Committee of Zhejiang Chinese Medical University (Approval No. 20230918-22).

**Fig. 1:**
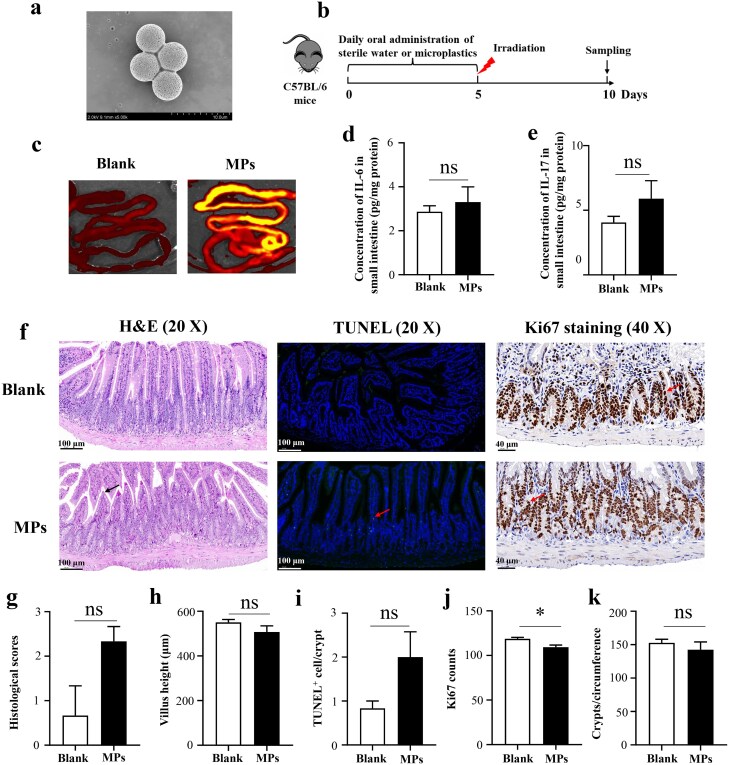
Effects of MPs on the small intestine of C57BL/6 mice. (a) Morphological characterizations of MPs; (b) Experimental overview; (c) Detection of fluorescent MPs particles in the small intestine; (d) IL-6 levels in the small intestine; (e) IL-17 levels in the small intestine; (f) Representative H&E-stained (scale bar = 100 μm, black arrows indicate histopathological changes), TUNEL-stained (scale bar = 100 μm, red arrows indicate TUNEL-positive cells), and Ki67 -stained (scale bar = 40 μm, red arrows indicate Ki67-positive cells) intestinal sections of various groups; (g) Histological scores based on H&E staining; (h) Quantification of villus height based on H&E staining; (i) Quantification of TUNEL-positive cells based on TUNEL immunofluorescence staining; (j) Quantification of Ki67-positive cells based on Ki67 immunohistochemical staining; (k) Quantification of surviving crypts per circumference based on Ki67 immunohistochemical staining. *P < 0.05, ns = no significance.

Fluorescent, single-molecule polystyrene microplastics (CAS: 55136 microplastics) with a 5 μm particle size were purchased from Sigma-Aldrich, Inc. The 5 μm size was selected to facilitate handling and to exclude nanoscale effects. Prior to use, MPs were sonicated and suspended in distilled water at the desired concentration. The particle surface morphology, as shown in Fig. 1a, was characterized by scanning electron microscopy.

### Total abdominal irradiation

Mice were anesthetized by intraperitoneal injection of sodium pentobarbital (50 mg/kg) and subjected to total abdominal irradiation (TAI) at doses of 4 Gy or 10 Gy using a Varian TrueBeam linear accelerator (Varian Medical Systems, Palo Alto, CA) at a rate of 2 Gy/min. During irradiation, lead shields protected the cervical, thoracic, and lower limb regions, leaving only the abdominal area exposed. A 6 MV X-ray beam was delivered with a source-to-surface distance of 100 cm, and a coincident light field was used to verify accurate positioning. The field uniformity was calibrated prior to each session, and mice were placed at a consistent distance from the radiation source. Five days after irradiation, animals were euthanized and small intestinal tissues were collected for subsequent analyses.

### MPs exposure

MPs were administered by oral gavage at 1.0 mg/kg body weight per day for five consecutive days. This dose was chosen to approximate human ingestion rates, estimated at 40.1 μg/kg/day for adults and 87.8 μg/kg/day for children [20]. Due to a 12.3-fold difference in surface area-to-body weight ratio between mice (0.0066 m^2^/0.02 kg) and humans (1.6 m^2^/60 kg), a 12.3-fold higher dose was administered to mice to approximate human exposure. Therefore, in this study, the MPs dose of 1.0 mg/kg/day (approximately 12.3 times 0.087) was used.

### Experimental design

#### Experiment I

As illustrated in Fig. 1b, seven-week-old C57BL/6 mice were randomly assigned to one of two pretreatment groups (*n* = 21 per group): the normal group and MPs exposure group. After the 5-day pretreatment, each group was further subdivided into three irradiation subgroups (*n* = 7 each): (i) Blank group (0 Gy TAI), (ii) 4 Gy subgroup (TAI with a total dose of 4 Gy), and (iii) 10 Gy subgroup (TAI with a total dose of 10 Gy). Five days after irradiation, all mice were euthanized and small intestinal tissues were harvested for analysis.

#### Experiment II

**Fig. 8:**
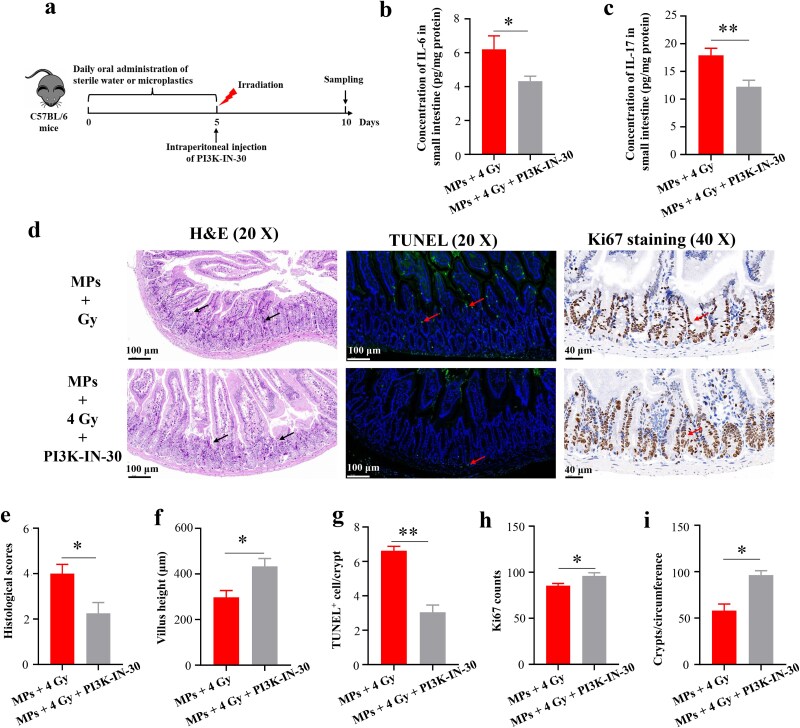
Effects of a PI3K inhibitor on MPs-sensitized radiation injury in the small intestine. (a) Schematics of the experimental timeline; (b) IL-6 levels in the small intestine; (c) IL-17 levels in the small intestine; (d) Representative H&E-stained (scale bar = 100 μm, black arrows indicate histopathological changes), TUNEL-stained (scale bar = 100 μm, red arrows indicate TUNEL-positive cells), and Ki67-stained (scale bar = 40 μm, red arrows indicate Ki67-positive cells) intestinal sections of various groups; (e) Histological scores based on H&E staining; (f) Quantification of villus height based on H&E staining; (g) Quantification of TUNEL-positive cells based on TUNEL immunofluorescence staining; (h) Quantification of Ki67-positive cells based on Ki67 immunohistochemical staining; (i) Quantification of surviving crypts per circumference based on Ki67 immunohistochemical staining. ^*^P < 0.05, ^**^P < 0.01.

As shown in Fig. 8a, C57BL/6 mice were first exposed to MPs for 5 days. MP-treated mice were then randomized into two intervention arms (*n* = 7 per arm): (i) MPs + 4 Gy group: received 4 Gy TAI and intraperitoneal injection of 10% DMSO; (ii) MPs + 4 Gy + P13K-IN-30 group: received 4 Gy TAI and intraperitoneal injection of 100 mg/kg P13K-IN-30 (dissolved in 10% DMSO). Five days postirradiation, mice were euthanized and small intestinal tissues were collected for downstream assays.

### Enzyme-linked immunosorbent assay

Small intestines were cleared of fecal contents and rinsed in sterile PBS (pH 7.4). Tissues were weighed and homogenized at 0.05 g per ml of lysis buffer using a high-speed homogenizer. Homogenates were centrifuged at 12 000 rpm for 10 min at 4°C, and the supernatants were collected. IL-6 and IL-17 levels were quantified with Multisciences enzyme-linked immunosorbent assay kits (Hangzhou, China) according to the manufacturer’s instructions. Total protein in each supernatant was measured by the micro-Bicinchoninic acid (BCA) method, and cytokine concentrations were expressed as pg cytokine per mg total protein.

### Pathological histology

Small intestines were harvested at designated time points postirradiation, flushed with ice-cold PBS, and a segment fixed overnight in 4% paraformaldehyde. Fixed tissues were dehydrated through an ethanol series, embedded in paraffin, and sectioned at 3 μm. Sections were stained with hematoxylin and eosin (H&E) and examined by light microscopy. Small intestinal injury was scored on a 0–5 scale adapted from Chang *et al.* [21]: 0: mucosa with normal villi; 1: subepithelial space development at the villous apex, often with capillary congestion; 2: scattered epithelial denudation at villous tips; 3: scattered epithelial denudation at villous tips; 4: epithelial shedding along both apex and mid-villi, with shortened and widened villi, and dilated capillaries; 5: complete villous destruction with lamina propria disintegration and ulceration.

### Ki67 immunohistochemistry

Paraffin-embedded small intestine sections were deparaffinized in xylene and rehydrated through a graded ethanol series to distilled water. Endogenous peroxidase activity was quenched by incubating sections in 3% hydrogen peroxide for 15 min. Antigen retrieval was carried out by pressure-cooking sections in sodium citrate buffer (pH 6.0) for 3 min, then allowing them to cool to room temperature. Non-specific binding was blocked with 5% bovine serum albumin for 30 min. Sections were incubated overnight at 4°C with rabbit anti-Ki67 primary antibody (Servicebio, CAS: GB111141; 1:800). The following day, they were incubated with HRP-conjugated secondary antibody (Servicebio, GB23303) at 37°C for 1 h. Signals were visualized using DAB substrate, and nuclei were counterstained with hematoxylin. Finally, slides were dehydrated, cleared, and mounted for light-microscopic analysis.

### TUNEL immunofluorescence

Apoptotic cells in the small intestine tissues were detected using a TUNEL Staining Kit (Servicebio, Wuhan, China). Briefly, paraffin-embedded sections were first permeabilized with 0.1% Triton X-100 for 20 min, then incubated at 37°C for 1 h with a labeling mixture of terminal deoxynucleotidyl transferase, deoxyuridine triphosphate (dUTP), and reaction buffer in a volume ratio of 2:5:50. Following incubation, slides were counterstained with DAPI and examined under a Leica fluorescence microscope. TUNEL-positive (green-fluorescent) cells were quantified in at least 30 well-oriented crypts per mouse.

### 4D-DIA quantitative proteomic analysis and data processing

Frozen small intestine tissue samples were retrieved and placed on ice. The study employed 4D-DIA quantitative proteomics to analyze protein profiles in these samples, following the protocol outlined in the previous work [7]. Briefly, thawed tissue samples were suspended in protein lysis buffer (8 M urea, 1% SDS) containing protease inhibitors, homogenized using a high-flux tissue grinder for three cycles of 40 s, and incubated on ice for 30 min with vortexing every 5 min. The samples were then centrifuged at 16 000 g for 30 minutes at 4°C. Protein concentrations in the supernatants were determined via BCA assay and confirmed by SDS-PAGE electrophoresis. For protein digestion, 100 μg of extracted protein was resuspended in 100 mM TEAB, reduced with 10 mM TCEP at 37°C for 60 min, and alkylated with 40 mM IAM for 40 min at room temperature in the dark. After centrifugation, the sample was resuspended in 100 mM TEAB and digested overnight with trypsin at a 1:50 ratio at 37°C. The resulting peptides were resolubilized with 0.1% TFA, desalted, and quantified.

Equal amounts of peptides were dried, resolubilized with 0.1% TFA, and analyzed using an EASY-nLC system coupled to a timsTOF Pro2 mass spectrometer (Bruker) at Majorbio Bio-Pharm Technology Co. Ltd (Shanghai, China). DIA data were acquired in DIA-PASEF mode with specific gradient and flow rate settings. Spectronaut (Version 14) was used to process DIA-PASEF data, correcting retention times using iRT standards. Quantification was based on six peptides per protein and three ions per peptide. The analysis parameters included: Protein FDR ≤0.01, Peptide FDR ≤0.01, Peptide Confidence ≥99%, and XIC width ≤75 ppm. Only proteins with unique peptides were considered for identification.

Bioinformatic analysis was conducted using the Majorbio Cloud platform (https://cloud.majorbio.com). Differentially expressed proteins (DEPs) were identified based on a fold change (FC) > 2 and *P*-value <0.05, calculated using the ‘*t*-test’ R package. Functional annotation of altered proteins was performed using KEGG pathway analysis (http://www.genome.jp/kegg/), with an adjusted *P*-value of <0.05 considered significant for enrichment. DEPs were further subjected to KEGG enrichment analysis.

### Statistical analysis

Statistical analysis was performed using GraphPad Prism version 8.0 (GraphPad Software Inc., San Diego, CA, USA). Comparisons between the blank and MPs groups, as well as between the MPs + 4 Gy and MPs + 4 Gy + PI3K-IN-30 groups, were conducted using two-tailed independent samples t-tests. Additionally, quantitative analyses comparing the blank, 4 Gy, and 10 Gy groups and the MPs, MPs + 4 Gy, and MPs + 10 Gy groups were performed using one-way analysis of variance followed by Dunnett’s post hoc test for multiple comparisons. Statistical significance was defined as *P* < 0.05.

## RESULTS

### Effects of MPs on the small intestine and its proteomics

As depicted in Fig. 1c, five days of oral MPs administration led to a significant accumulation of MPs in the small intestines of C57BL/6 mice. But overall, histological changes were minimal, likely reflecting the brief exposure. However, the impact of MPs on the small intestine was negligible, probably because the exposure duration was so short. Figure 1d and e demonstrates that MPs exposure did not significantly alter IL-6 and IL-17 levels in the small intestine. Although H&E and TUNEL staining revealed modest small intestinal tissue damage, there were no significant differences in pathological scores, villus height or numbers of apoptotic cells (Fig. 1f–i). In addition, Ki67 staining showed no obvious alteration in surviving crypts per circumference, but a clear reduction in proliferative activity of intestinal epithelium cells (Fig. 1f and j). In summary, short-term MPs exposure induced only mild histological alterations, with the limited duration likely constraining injury severity.

**Fig. 2:**
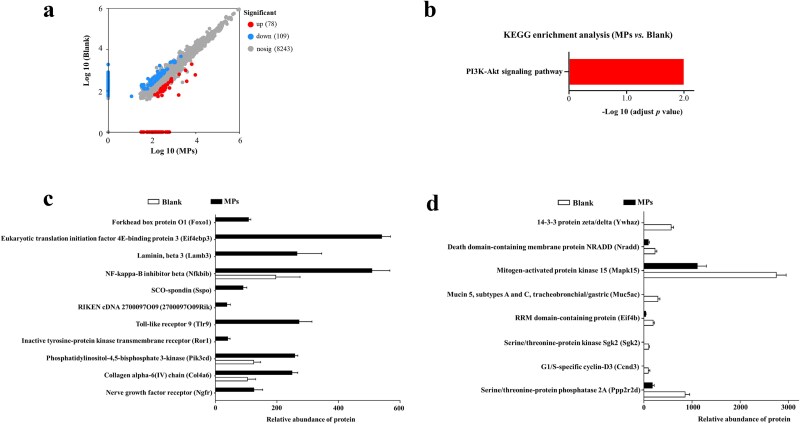
Effects of MPs on small intestine proteomics. (a) Volcano plot displaying *P* values and log FCs of intestinal proteins in blank versus MPs-treated mice, red dots denote proteins upregulated by MPs, light-blue dots denote proteins downregulated by MPs; (b) KEGG Level 3 functional classification of DEPs between blank and MPs groups; (c) Intestinal proteins significantly upregulated by MPs that map to the ‘PI3K-Akt signaling’ pathway; (d) Intestinal proteins significantly downregulated by MPs that map to the ‘PI3K-Akt signaling’ pathway.

Proteomic analysis of small intestinal tissue identified 187 DEPs between blank and MP-exposed mice, with 78 upregulated and 109 downregulated in the MPs group (Fig. 2a). KEGG enrichment analysis revealed significant overrepresentation of the ‘PI3K–Akt signaling pathway’ (adjusted *P <* 0.05) (Fig. 2b). Within this pathway, 19 DEPs were detected (11 upregulated and 8 downregulated in MPs group; Fig. 2c and d). The upregulated proteins, among them Nfkbib, Tlr9, Pik3cd, Col4a6, and Ngfr, are chiefly linked to inflammatory processes (Fig. 2c).

### Effects of irradiation on the small intestine and its proteomics in C57BL/6 mice

**Fig. 3:**
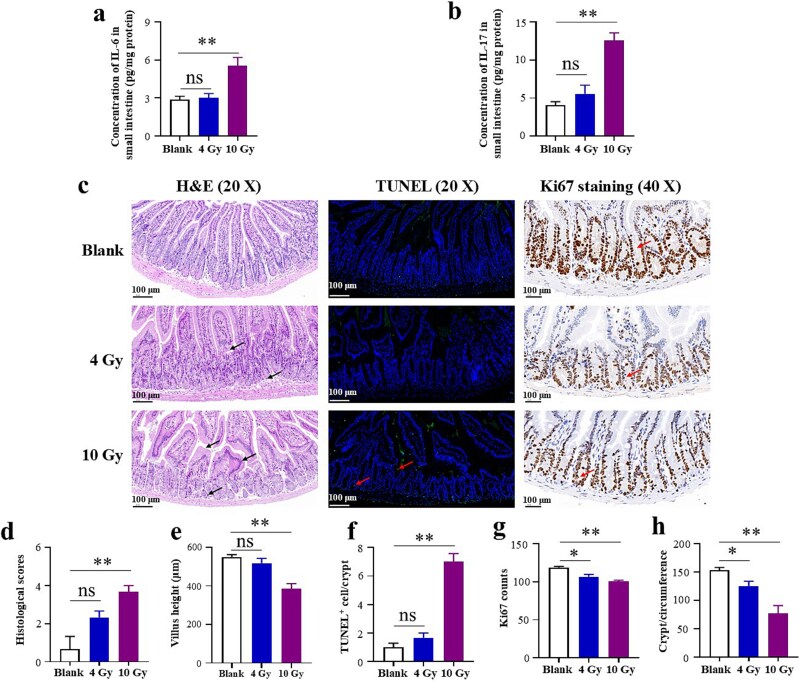
Effects of irradiation on the small intestine of C57BL/6 mice. (a) IL-6 levels in the small intestine; (b) IL-17 levels in the small intestine; (c) Representative H&E-stained (scale bar = 100 μm, black arrows indicate histopathological changes), TUNEL-stained (scale bar = 100 μm, red arrows indicate TUNEL-positive cells), and Ki67 -stained (scale bar = 40 μm, red arrows indicate Ki67-positive cells) intestinal sections of various groups; (d) Histological scores based on H&E staining; (e) Quantification of villus height based on H&E staining; (f) Quantification of TUNEL-positive cells based on TUNEL immunofluorescence staining; (g) Quantification of Ki67-positive cells based on Ki67 immunohistochemical staining; (h) Quantification of surviving crypts per circumference based on Ki67 immunohistochemical staining. ^*^P < 0.05, ^**^P < 0.01, ns = no significance.

As shown in Fig. 3, irradiation produced dose-dependent damage to the small intestine of C57BL/6 mice. A 4 Gy dose had minimal impact, with no significant alterations in IL-6 or IL-17 expression (Fig. 3a and b). In contrast, 10 Gy irradiation markedly upregulated IL-6 and IL-17 levels (Fig. 3a and b). H&E and TUNEL staining corroborated these results: 4 Gy induced only mild villus widening, without significant differences in pathology scores, villus height or apoptotic cell counts (Fig. 3c–f). Conversely, 10 Gy caused severe villus injury-characterized by epithelial shedding and lamina propria disintegration-accompanied by significantly higher pathology scores, lower villus height, and increased apoptosis (Fig. 3c–f). Moreover, Ki67 staining demonstrated that both irradiation doses significantly diminished epithelial cell proliferation and surviving crypts per circumference in the small intestine (Fig. 3c and g).

**Fig. 4:**
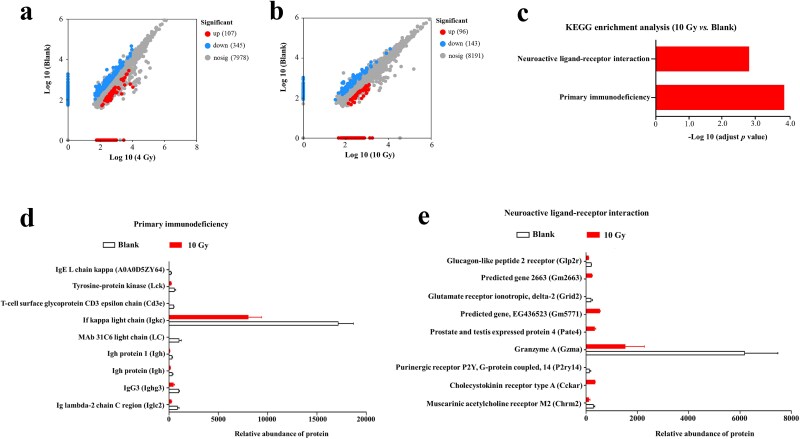
Effects of irradiation on small intestinal proteomics in C57BL/6 mice. (a) Volcano plot displaying *P*-values and log FCs of intestinal proteins between blank and 4 Gy groups, red dots denote proteins upregulated by 4 Gy, blue dots denote proteins downregulated by 4 Gy; (b) Volcano plot showing the *P*-values and log FCs of intestinal proteins between blank and 10 Gy groups, red dots denote proteins upregulated by 10 Gy, blue dots denote proteins downregulated by 10 Gy; (c) KEGG Level 3 functional classification of DEPs between blank and 10 Gy groups; (d) DEPs mapping to the ‘Primary immunodeficiency’ pathway in the Blank versus 10 Gy comparison; (e) DEPs mapping to the ‘Neuroactive ligand-receptor interaction’ pathway in the Blank versus 10 Gy comparison.

Proteomic analysis further demonstrated a dose-dependent effect of irradiation on small intestinal protein expression. As shown in Fig. 4a and b 4 Gy irradiation yielded 452 DEPs, whereas 10 Gy produced 239 DEPs. KEGG enrichment analysis revealed no significantly enriched pathways among the 4 Gy-induced DEPs. In contrast, the DEPs induced by 10 Gy irradiation were significantly enriched in the ‘primary immunodeficiency’ and ‘neuroactive ligand–receptor interaction’ pathways (Fig. 4c). Nine DEPs downregulated by 10 Gy mapped to the ‘primary immunodeficiency’ pathway (Fig. 4d), indicating potential disruption of immune function. Likewise, nine DEPs were associated with the ‘neuroactive ligand-receptor interaction’ pathway, with four upregulated and five downregulated in response to 10 Gy irradiation (Fig. 4e). These enriched pathways likely contribute to the mechanisms underlying RIII.

### Effects of irradiation on the small intestine of C57BL/6 mice preexposed to MPs

**Fig. 5:**
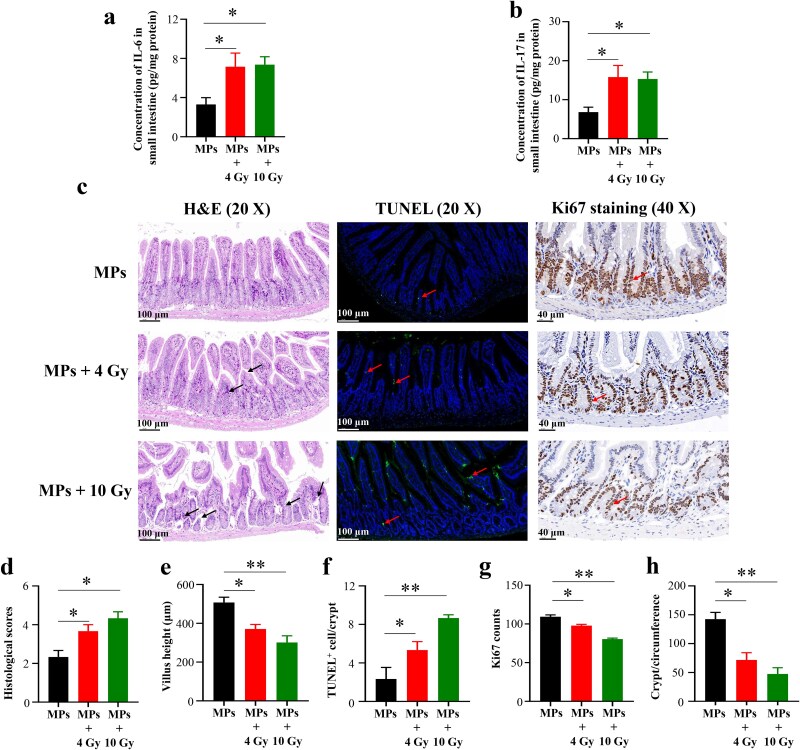
Effects of irradiation on the small intestine of C57BL/6 mice preexposed to MPs. (a) IL-6 concentration in the small intestine; (b) IL-17 concentration in the small intestine; (c) Representative H&E-stained (scale bar = 100 μm, black arrows indicate histopathological changes), TUNEL-stained (scale bar = 100 μm, red arrows indicate TUNEL-positive cells), and Ki67-stained (scale bar = 40 μm, red arrows indicate Ki67-positive cells) intestinal sections of various groups; (d) Histological scores based on H&E staining; (e) Quantification of villus height based on H&E staining; (f) Quantification of TUNEL-positive cells based on TUNEL immunofluorescence staining; (g) Quantification of Ki67-positive cells based on Ki67 immunohistochemical staining; (h) Quantification of surviving crypts per circumference based on Ki67 immunohistochemical staining. ^*^P < 0.05, ^**^P < 0.01.

To assess how prior MPs exposure alters radiation response, C57BL/6 mice were first given MPs and then irradiated with 4 Gy or 10 Gy. Both doses produced significantly greater intestinal injury in MPs-exposed mice, as shown by upregulated IL-6 and IL-17 in small intestinal tissue (Fig. 5a and b). H&E staining revealed that 4 Gy further disrupted villus architecture, whereas 10 Gy caused extensive villus and lamina propria disintegration (Fig. 5c–e). Correspondingly, histological scores and TUNEL assays demonstrated dose-dependent increases in tissue injury and apoptotic cell numbers (Fig. 5c–f). Finally, Ki67 staining showed a marked, dose-dependent reduction in epithelial proliferation and surviving crypts per circumference (Fig. 5c and g), indicating that short-term MPs exposure sensitizes the small intestine to radiation-induced damage.

### Effects of irradiation on the small intestinal proteome in C57BL/6 mice preexposed to MPs

**Fig. 6:**
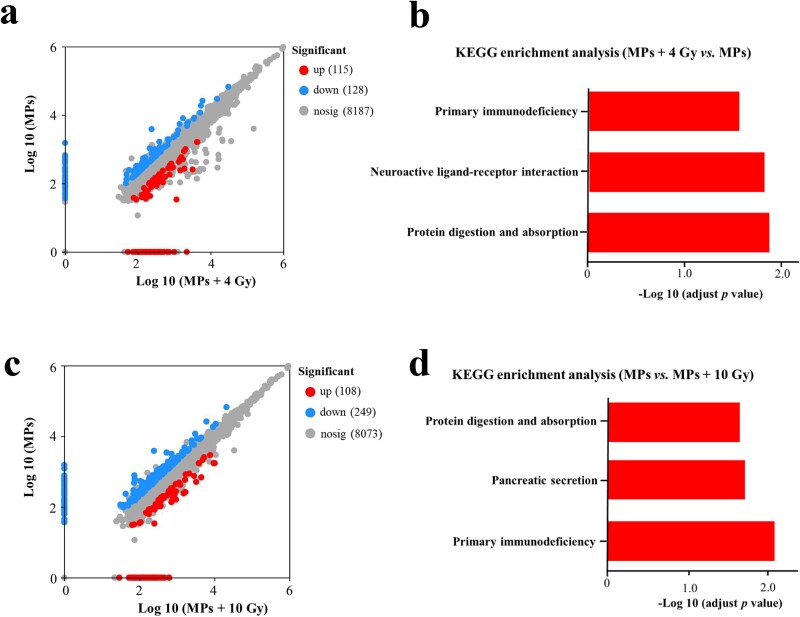
Effects of irradiation on the small intestine proteomics in C57BL/6 mice preexposed to MPs. (a) Volcano plot displaying *P*-values and log FCs of intestinal proteins between MPs and MPs + 4 Gy groups, red dots indicate proteins upregulated by irradiation, blue dots indicate proteins downregulated by irradiation; (b) KEGG Level 3 functional classification of DEPs between MPs and MPs + 4 Gy groups; (c) Volcano plot displaying *P*-values and log FCs of intestinal proteins between MPs and MPs + 10 Gy groups, red dots indicate proteins upregulated by irradiation, blue dots indicate proteins downregulated by irradiation; (d) KEGG level 3 functional classification of DEPs between MPs and MPs + 10 Gy groups.

In mice preexposed to MPs, 4 Gy irradiation induced pronounced intestinal injury and substantially remodeled the small intestinal proteome. Comparison of the MPs and MPs + 4 Gy groups identified 243 DEPs, with 115 upregulated and 128 downregulated in the irradiated group (Fig. 6a). KEGG enrichment analysis revealed three significantly enriched pathways among these DEPs: ‘Primary immunodeficiency’, ‘Neuroactive ligand–receptor interaction’, and ‘Protein digestion and absorption’ (Fig. 6b). The DEPs involved in these KEGG pathways were shown in Supplementary Fig. S1.

In MPs preexposed mice, 10 Gy irradiation significantly altered the small intestinal proteome, yielding 357 DEPs (108 upregulated and 249 downregulated, Fig. 6c). KEGG enrichment analysis identified three significantly overrepresented pathways: ‘Primary immunodeficiency’, ‘Pancreatic secretion’, and ‘Protein digestion and absorption’ (Fig. 6d). The ‘Primary immunodeficiency’ pathway was enriched again, driven by downregulation of its constituent proteins, mirroring the pattern seen in non-MP-exposed mice subjected to 10 Gy (Supplementary Fig. S2). Furthermore, the ‘Protein digestion and absorption’ pathway overlapped extensively with the ‘Pancreatic secretion’ pathway, with both dominated by digestive enzymes.

Overall, whereas 4 Gy alone failed to enrich any pathways in normal mice, the same dose in MPs preexposed mice triggered significant enrichment of three key pathways. In MPs-exposed mice, 10 Gy irradiation not only recapitulated the immunodeficiency signature observed at 4 Gy but also uniquely implicated exocrine pancreatic pathways. This highlights an exacerbated vulnerability in both immune and digestive functions within the irradiated, MPs-sensitized intestine.

### Effects of preexposure to MPs on RIII in C57BL/6 mice

**Fig. 7:**
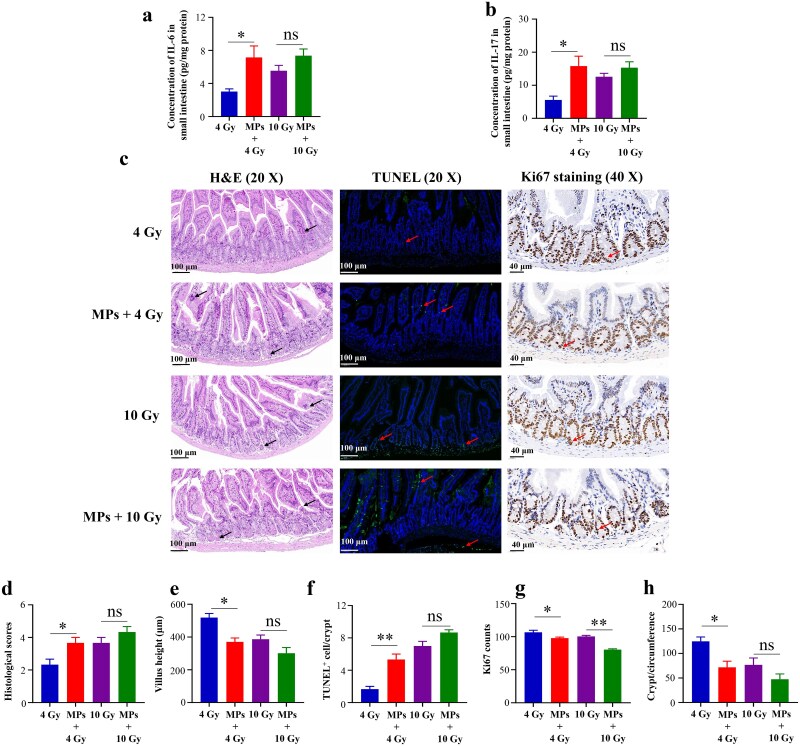
Effects of pre-MPs exposure on RIII in C57BL/6 mice. (a) IL-6 concentration in the small intestine; (b) IL-17 concentration in the small intestine; (c) Representative H&E-stained (scale bar = 100 μm, black arrows indicate histopathological changes), TUNEL-stained (scale bar = 100 μm, red arrows indicate TUNEL-positive cells), and Ki67 -stained (scale bar = 40 μm, red arrows indicate Ki67-positive cells) intestinal sections of various groups; (d) Histological scores based on H&E staining; (e) Quantification of villus height based on H&E staining; (f) Quantification of TUNEL-positive cells based on TUNEL immunofluorescence staining; (g) Quantification of Ki67-positive cells based on Ki67 immunohistochemical staining; (h) Quantification of surviving crypts per circumference based on Ki67 immunohistochemical staining. *P < 0.05, **P < 0.01, ns = no significance.

To further investigate the impact of prior MPs exposure on RIII, we compared the effects of radiation alone with those of combined radiation and MPs exposure. At a 4 Gy radiation dose, prior MPs exposure significantly exacerbated RIII in C57BL/6 mice, as evidenced by increased intestinal IL-6 and IL-17 levels, elevated pathological scores, increased TUNEL^+^ cells, and reduced villus height, Ki67^+^ cells, and crypts per circumference (Fig. 7). In contrast, at a 10 Gy radiation dose, prior MPs exposure only marginally aggravated radiation-induced damage, with a significant reduction in Ki67^+^ cells but no notable statistical differences in other markers (Fig. 7). This discrepancy likely arises from the dose-dependent effects of radiation on intestinal damage. At higher doses, severe radiation-induced injury might obscure the additional effects of prior MPs exposure.

In addition, small intestinal proteomics revealed the effects of prior MPs exposure on radiation-induced alterations in intestinal proteins. At a 4 Gy radiation dose, prior MPs exposure significantly profoundly upregulated 217 proteins and downregulated 113 proteins (Supplementary Fig. S3a). These DEPs were enriched in the ‘Mineral absorption’ and ‘Neuroactive ligand-receptor interaction’ pathways (Supplementary Fig. S3b). At a 10 Gy radiation dose, prior MPs exposure significantly upregulated 69 proteins and downregulated 146 proteins (Supplementary Fig. S3c). However, the DEPs identified between MPs + 10 Gy and 10 Gy groups showed no significant pathway enrichment.

### Effects of a PI3K inhibitor on 4 Gy irradiation-induced small intestinal injury in C57BL/6 mice preexposed to MPs

To clarify how MPs exposure amplified small intestinal sensitivity to radiation, we treated MPs preexposed C57BL/6 mice with the selective PI3K inhibitor PI3K-IN-30 before administering 4 Gy irradiation. PI3K-IN-30 markedly attenuated the MP-exacerbated damage, as shown by decreased IL-6 and IL-17 levels, lower histopathological scores, reduced apoptosis, and restored villus height, Ki67-positive epithelial proliferation, and surviving crypts per circumference (Fig. 8). Together, these data demonstrated that PI3K signaling is a critical mediator of MPs-sensitized radiation injury in the small intestine.

## DISCUSSION

Both irradiation exposure and MPs contamination pose significant environmental and health risks. Although prior investigations have largely examined radiation and MP exposure separately [22, 23], their combined effects remain poorly characterized. Here, we show that short-term MPs exposure markedly increases the small intestine’s vulnerability to radiation damage in C57BL/6 mice.

A recent study by Chen *et al.* [24] demonstrated that MPs exposure deteriorated RIII in mice via alterations in gut microbiota, with the small intestine more affected than the colon. While our work confirms that MP exposure worsens RIII, it diverges from Chen’s in three key ways: (i) Chen *et al.* emphasized microbiome shifts, whereas we interrogated proteomic changes in the small intestine; (ii) We compared both low-dose (4 Gy) and high-dose (10 Gy) exposures, placing particular emphasis on the subtler effects of low-dose radiation that better reflect clinical settings; (iii) Rather than focusing solely on exacerbation of established RIII, we highlighted how MPs preexposure increases intrinsic tissue sensitivity to radiation. These distinctions deepen our mechanistic understanding of how MPs modulate RIII and unveil novel insights.

Brief MP exposure alone caused minimal small intestinal injury, likely due to the short duration. However, proteomic analysis revealed altered protein expression, with the PI3K-Akt signaling pathway the only significantly enriched pathway. Given its pivotal role in regulating cell growth, proliferation, and survival [25], these data implicate MPs-induced dysregulation of PI3K-Akt signaling. Existing literature presented divergent findings on PI3K-Akt pathway modulation by MPs, with some studies demonstrating inhibition of PI3K-Akt pathway [26, 27] and others reporting activation [28, 29]. MPs could induce the dysregulation of PI3K-Akt pathway through multiple mechanisms, including physical barrier disruption [29], oxidative stress induction [30, 31], inflammatory response activation [32, 33], and its direct chemical toxicity [26]. To confirm this mechanism, we treated MPs preexposed mice with the PI3K inhibitor PI3K-IN-30, which normalized PI3K activity and prevented MPs-driven sensitization of the small intestine to radiation-induced injury by modulating the PI3K-Akt axis. Moreover, prior studies have also demonstrated that pharmacological inhibition of the PI3K-Akt pathway mitigated RIII in experimental models [34, 35]. The PI3K-Akt pathway inhibitor LY294002 was also reported to inhibit the radioprotective effects in connexin 43-overexpressing intestinal epithelial cells [36]. The previous research and our study demonstrated variable effects of PI3K-Akt inhibitors on radiation damage. The variability likely arises from differences in basal PI3K-Akt pathway activity. Specifically, exposure to MPs could upregulate PI3K-Akt signaling pathway, while connexin 43 suppressed this pathway.

In our radiation experiments, 4 Gy caused minimal intestinal damage, whereas 10 Gy induced pronounced injury. Proteomic analysis revealed that only 10 Gy—in contrast to 4 Gy—significantly enriched the ‘primary immunodeficiency’ and ‘neuroactive ligand–receptor interaction’ pathways. The ‘primary immunodeficiency’ pathway was essential for maintaining immune homeostasis [37], and this data indicated that irradiation impairs this pathway, leading to immune dysfunction [38]. Such dysfunction could intensify inflammation, hinder tissue repair, and increase susceptibility to infection, all of which exacerbated radiation enteropathy [39]. Likewise, the ‘neuroactive ligand-receptor interaction’ pathway regulated gut motility [40] and immune responses [41], both of which were disrupted by irradiation [42, 43]. Perturbation of this pathway may further worsen intestinal injury by impairing motility and amplifying inflammatory responses. These proteomic findings therefore offered new insight into the mechanisms driving RIII.

We next examined how prior MPs exposure influences RIII. Similar to Chen *et al.* [24], who reported MPs-driven exacerbation of established RIII, we demonstrated that prior MPs exposure exacerbated RIII in mice subjected to 4 Gy irradiation. Furthermore, our extended analysis revealed that prior MPs exposure sensitized the small intestine to irradiation. In normal mice, 4 Gy caused minimal injury, whereas the same dose produced severe damage in MPs preexposed mice. Both 4 Gy and 10 Gy in MPs preexposed mice significantly enriched three KEGG pathways—‘primary immunodeficiency’, ‘neuroactive ligand–receptor interaction’, and ‘protein digestion and absorption’—all of which were closely linked to RIII. Notably, the ‘protein digestion and absorption’ pathway, which breaks down dietary proteins into amino acids and peptides essential for tissue repair, enzyme production, and immune defense [44], was also enriched in mice receiving 13 Gy in a previous study [45]. Since irradiation damages intestinal villi and crypts—key sites for nutrient uptake [46]—disruption of this pathway may exacerbate malnutrition, impede mucosal repair, and weaken immune responses, thereby prolonging recovery and increasing vulnerability to further injury and infection. Therefore, the ‘protein digestion and absorption’ pathway might be involved in RIII in the mice preexposed to MPs.

Nonetheless, this study has several limitations that should be acknowledged. First, the exclusive use of male mice may constrain the generalizability of the findings to female populations. Second, the investigation was confined to 5 μm polystyrene microplastics and did not assess other particle sizes or polymer types. Third, while PI3K inhibitors were examined specifically for their role in MPs-aggravated RIII, their independent effects on RIII were not evaluated. Finally, as pentobarbital provided no analgesia or muscle relaxation, its use as a sole anesthetic might lead to unmitigated pain during TAI process.

## CONCLUSION

In conclusion, this study demonstrated that prior MPs exposure markedly increases the mouse intestine’s susceptibility to radiation-induced injury via dysregulation of the PI3K-Akt pathway. While these findings deepen our mechanistic insight, three crucial next steps are needed: (i) confirm these effects in human cohorts or organoid models; (ii) Systematically assess interactions between MPs and other radiation types (e.g. neutrons, UV-C); (iii) Define the specific intestinal protein targets and metabolic circuits through which PI3K-Akt signaling mediates MPs-sensitized injury. Taken together, our results underscore the importance of minimizing MPs exposure in cancer patients undergoing radiotherapy and more broadly illustrate how MPs can synergize with other environmental hazards to amplify tissue damage.

## Supplementary Material

Supplementary_Figures_rraf046

## Data Availability

Data will be made available on request.
