# Supplementary material for: Prior exposure to microplastics heightened the susceptibility of small intestine to radiation-induced injury in C57BL/6 mice
Source: J Radiat Res. 2025 Jul 26;66(5):473–85. doi: 10.1093/jrr/rraf046 (PMC12460044; doi:10.1093/jrr/rraf046)
Supplement: Supplementary_Figures_rraf046 [file supplementary_figures_rraf046.pdf]

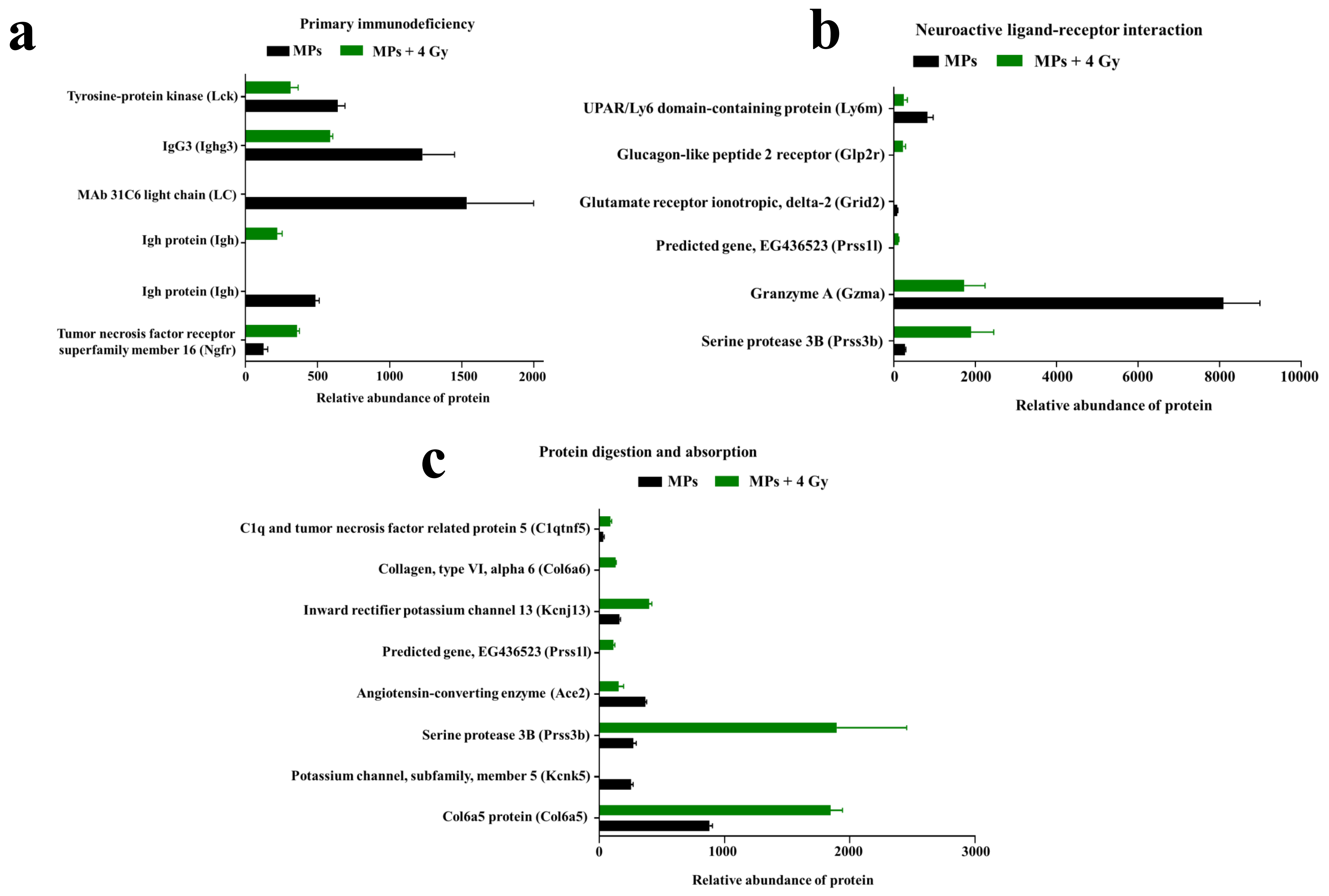

Fig. S1 Effects of 4 Gy irradiation on the small intestine proteomics in C57BL/6 mice pre-exposed to MPs. (a) DEPs mapping to the "Primary immunodeficiency" pathway between MPs and MPs + 4 Gy groups; (b) DEPs mapping to the "Neuroactive ligand-receptor interaction" pathway between MPs and MPs + 4 Gy groups; (c) DEPs mapping to the "Protein digestion and absorption" pathway between MPs and MPs + 4 Gy groups.

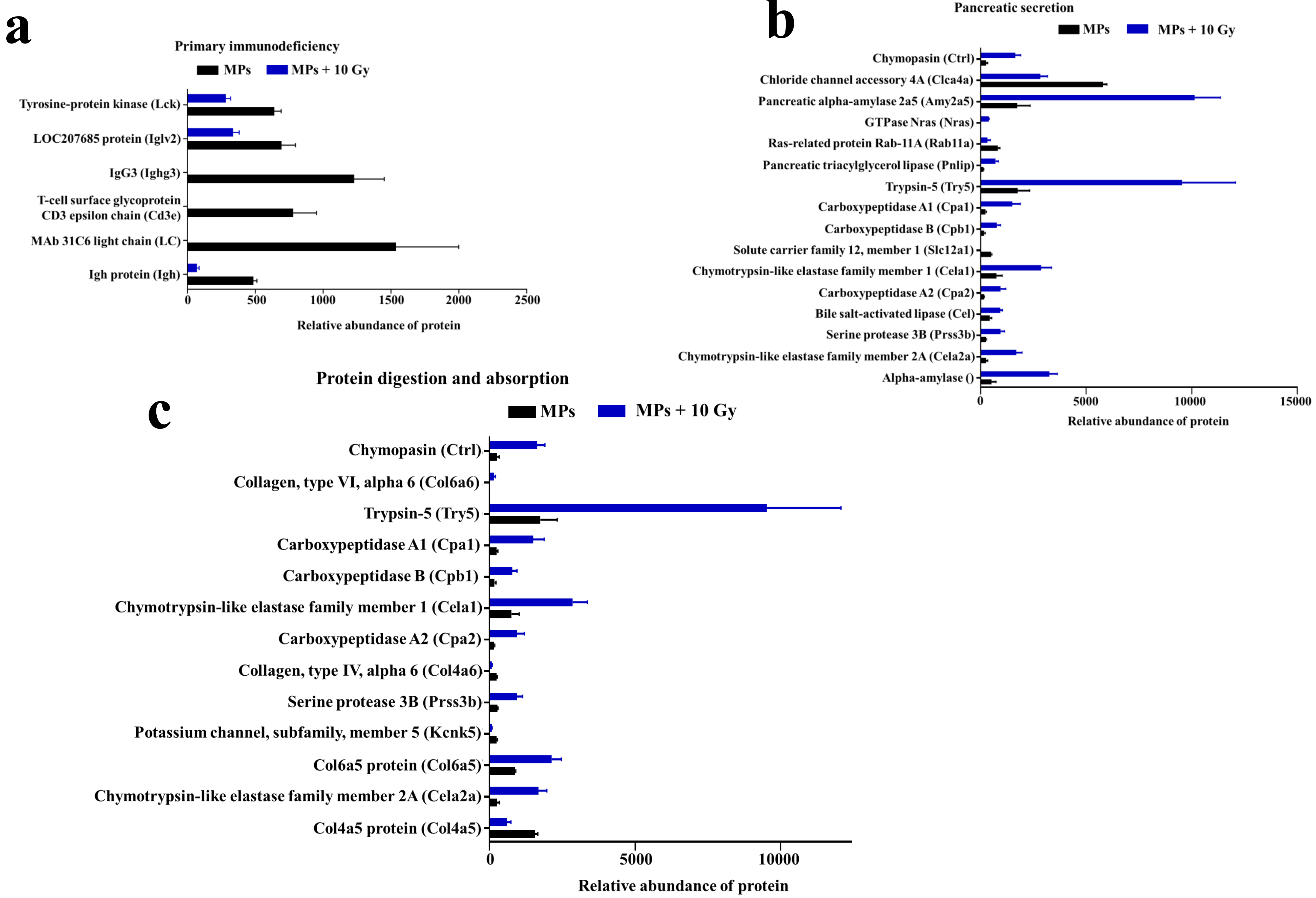

Fig. S2 Effects of 10 Gy irradiation on the small intestine proteomics in C57BL/6 mice pre-exposed to MPs. (a) DEPs mapping to the “Primary immunodeficiency” pathway between MPs and MPs + 10 Gy groups; (b) DEPs mapping to the “Pancreatic secretion” pathway between MPs and MPs + 10 Gy groups; (c) DEPs mapping to the “Protein digestion and absorption” pathway between MPs and MPs + 10 Gy groups.

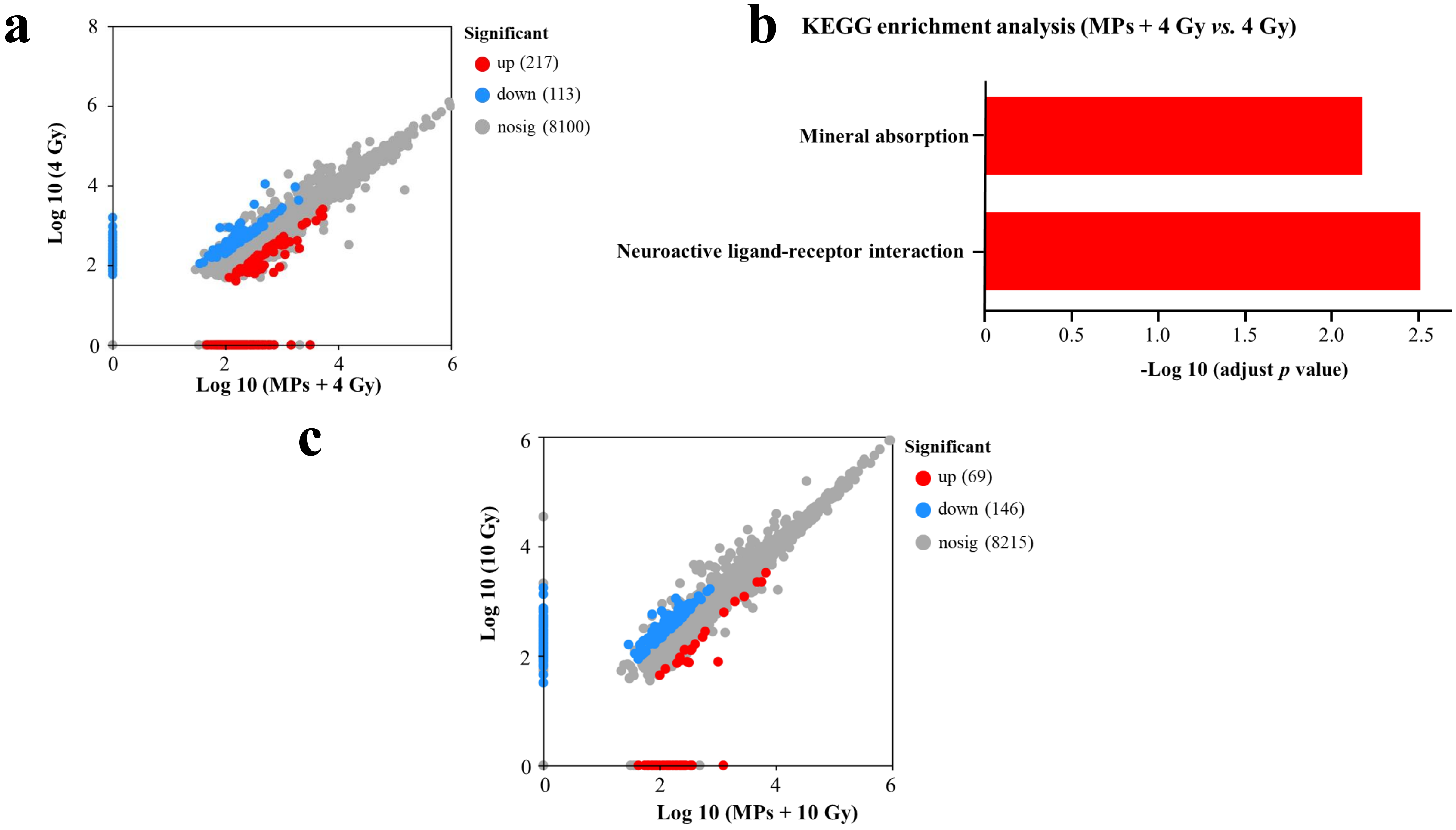

Fig. S3 (a) Volcano plot displaying p-values and log fold changes of intestinal proteins between MPs + 4 Gy and 4 Gy groups, red dots indicate upregulated proteins in MPs + 4 Gy irradiation, blue dots indicate downregulated proteins in MPs + 4 Gy; ; (b) KEGG level 3 functional classification of differentially expressed proteins between MPs + 4 Gy and 4 Gy groups; (c) Volcano plot displaying p-values and log fold changes of intestinal proteins between MPs + 10 Gy and 10 Gy groups, red dots indicate upregulated proteins in MPs + 10 Gy irradiation, blue dots indicate downregulated proteins in MPs + 10 Gy.
